# A Qualitative Evaluation of a Mother and Child Center Providing Psychosocial Support to Newly Arrived Female Refugees in a Registration and Reception Center in Germany

**DOI:** 10.3390/ijerph18094480

**Published:** 2021-04-23

**Authors:** Catharina Zehetmair, David Kindermann, Inga Tegeler, Cassandra Derreza-Greeven, Anna Cranz, Hans-Christoph Friederich, Christoph Nikendei

**Affiliations:** Center for Psychosocial Medicine, Department of General Internal Medicine and Psychosomatics, Heidelberg University Hospital, 69115 Heidelberg, Germany; david.kindermann@med.uni-heidelberg.de (D.K.); ingategeler@gmx.de (I.T.); Cassandra.Derreza-Greeven@med.uni-heidelberg.de (C.D.-G.); Anna.Cranz@med.uni-heidelberg.de (A.C.); Hans-Christoph.Friederich@med.uni-heidelberg.de (H.-C.F.); Christoph.Nikendei@med.uni-heidelberg.de (C.N.)

**Keywords:** psychosocial health care, refugees, female, asylum seekers, protective shelter

## Abstract

Female refugees are frequently exposed to sexualized, gender-based violence and harassment before, during, and after their flight. Yet female refugee-specific care and protection needs are rarely addressed in host countries. This study aimed to evaluate a mother and child center (MUKI) for female refugees in a reception and registration center in Germany. In 2017, we conducted semi-structured qualitative interviews with 16 female refugees attending the MUKI and with its five main staff members. We asked the participants about the MUKI’s relevance, encountered difficulties, and suggestions for improvement. The interviewees appreciated the MUKI’s sheltered environment, care services, and socializing opportunities, as well as its women-only concept. Overall, the participants saw overexertion, social engagement-related difficulties, and the MUKI’s noisy environment as key attendance barriers. Interviewed staff primarily reported problems regarding the working conditions, including the high staff and attendee turnover and low general service awareness. The participants advocated an expansion of the MUKI program. The MUKI project underlines that providing newly arrived, vulnerable female refugees with sheltered surroundings and psychosocial services is an essential step toward addressing female refugees’ specific care needs.

## 1. Introduction

### 1.1. Refugee Women

Some 70.8 million people worldwide were seeking safety and stability because of war, human rights violations, persecution, and economic hardship in late 2018 [1]. Estimates suggest that at least 48% are forcibly displaced women and girls [1]. This highly vulnerable refugee group is significantly affected by pre-, peri-, and post-migratory trauma and stress factors, leading to severe psychological burden [2]. Sexualized and gender-based violence are among the main reasons women and girls flee their home countries [3]. Forced marriage, family honor-related violence, genital mutilation, rape, and resistance to and or transgression of gender-discriminatory religious practices, cultural traditions, or legislation are just some examples of what females experience before and after armed conflicts [4,5,6]. During their flight, women and children are particularly vulnerable to sexual and other exploitation forms, including labor exploitation, trafficking, discrimination, and extortion [3,6,7,8]. A representative survey of 639 female refugees living in German shelters revealed that a significantly higher number of women from African countries had traveled to Germany alone than women fleeing from Syria, Afghanistan, and Iraq. Of these, 81% were accompanied by minors, 2.6% of whom had been born during flight [4].

In 2019, women accounted for 43% of all asylum applications in Germany, while 22% of all asylum applications were for young children under one year of age [9]. The European Union Directive (2013/33/EU; Chapter IV, Article 21) states that (unaccompanied) minors, pregnant women, and single parents with minors are the most vulnerable individuals among displaced populations. Many female refugees still experience discrimination and abuse, including sexual harassment and psychological or physical violence, in their host or destination countries [4,10]. Studies have shown that female refugees are more likely to experience psychological stress and have a higher risk of developing mental illness than male refugees [11,12,13,14]. Pregnant and postpartum refugees are also more likely to have high-risk pregnancies, abnormal neonatal birth parameters, and show more postpartum depression [15,16,17]. Female refugees often have limited health and psychosocial care access due to structural factors, stigmatization fears, low self-esteem, and shame [4,14]. Deacon and Sullivan [18] found that 42% of the refugee women preferred a female doctor in their sample. Although the need for gender-sensitive services and gender-specific access to health care is recognized, offers are still sparse [4,6].

### 1.2. Programs Addressing Specific Needs of Refugee Women

Internationally, some programs address refugee women’s specific needs in their home or host countries. In 2019, Hammer et al. [19] released a review about nineteen programs worldwide addressing the needs of internally displaced and refugee women and girls: for example, The Citizen Charter Afghanistan Project is aimed at developing better infrastructures (clean water, electricity, roads, and irrigation), health care, and education to communities across Afghanistan as well as sensitizing communities to the challenges faced by women (e.g., gender-based violence). The Development Response to Displacement Impacts Project (DRDIP, Horn of Africa, and Kenya) and the Jordan Emergency Health Project were primarily concerned with psychoeducation and prevention of sexual abuse and gender-based violence. The Piloting Delivery of Justice Sector Services to Poor Jordanians and Refugees in Host Communities project developed and implemented training and awareness campaigns on women and children’s needs. The IDP Living Standards and Livelihoods Project in Azerbaijan established women-only meetings to ensure their representation in community investment decisions [19]. The Hacettepe University Women’s Research and Implementation Center has been operating a women’s health counseling center in Turkey since 2015. It provides sexual and reproductive health care and family planning services, gender-based violence prevention offers, counseling, and leisure activities, such as yoga and language courses [20]. Khamphakdy-Brown et al. [21] introduced an empowerment program to prevent domestic violence, offering psychoeducation, workshops, home visits, counseling, and female shelters. Recently, Sabri et al. [22] published an article introducing a web-based application called ‘weWomen’, including risk assessments and safety planning for immigrant and refugee women facing intimate partner violence. Furthermore, the Utah Refugee Service Office offers refugee women workshops to increase their self-efficacy and management of their and their families’ health [23]. In the Netherlands, refugee women can attend special bicycle workshops to improve their mobility, access, and skills and increase their social engagement [24].

Regarding refugee camp settings, the humanitarian aid organization Médecins Sans Frontières has established a mental health clinic for female survivors of sexual violence in a Ugandan refugee camp [25]. The provision of better living conditions, such as access to energy and security teams, has been identified as a critical factor in addressing sexual and gender-based violence in refugee camps in developing countries [26]. For example, the Kenyan refugee camp of Kakuma has implemented a gender program: security units monitor women’s safety, and there are safe spaces for injured or vulnerable women [27]. They have also established educational programs, including scholarships and grants, and an all-girls boarding school. Empowerment assistance programs, human rights and gender issue workshops, sewing, cooking, knitting, nursing, and computer or electrical repair training courses are also offered [27]. In Cox’s Bazar refugee camps, primarily sheltering Rohingya refugees in Bangladesh, 52 safe access points and prevention programs, including engagement and empowerment interventions, have been set up for females [28]. Stark et al.’s review [29] of safe spaces programs for women and girls in the Democratic Republic of the Congo, Ethiopia, Uganda, Tanzania, Kenya, Bangladesh, and Pakistan found improvements in psychosocial well-being, social support, and attitudes toward rites of passage. Krause [30] highlights that flight can empower women by giving them more agency and choice. The women’s sense of empowerment is fostered by confidence-building activities, which are often offered by humanitarian relief organizations.

### 1.3. Study Concept

While there are special programs and initiatives geared toward improving refugee women’s safety and empowerment worldwide, the literature often describes shortfalls in addressing safety and gender needs in shelters or refugee camps leading to refugee women’s disempowerment [30]. In Germany, separate housing units and sanitary facilities, or simply lockable rooms and showers, are virtually non-existent [31]. Schouler-Ocak and Kurmeyer [4] point out that female refugee accommodation conditions in the destination or host country facilitate discrimination and sexualized assaults and discourage female refugees from articulating their needs and difficulties. Shelters in centers for newly arrived refugees specifically designed to safeguard female refugees are an exception in Germany [31]. The German Red Cross, Rhine-Neckar/Heidelberg division, has operated a low-threshold, psychosocial care service for newly arrived pregnant refugees and female refugees with children in the Heidelberg-Kirchheim reception and registration center in Germany since 2016 to address this issue. The operated mother–child center (MUKI) is a female-only space offering women and their children a sheltered and friendly environment to socialize, find mutual support, and receive childcare offers. It aims to strengthen psychosocial resources and offers German language, knitting, and cooking classes as well as monthly community cooking evenings focusing on traditional dishes from different cultures. In addition, the MUKI has weekly consultations regarding asylum procedures and midwifery care. Opening Monday through Friday between ten and one p.m., the MUKI’s services are open to all refugee women and their children.

This study aimed to evaluate the female refugees’ and staff’s experiences of the MUKI psychosocial care services for female refugees and their children. The interviews with the female refugees focused on (1) their motives for attending the MUKI, (2) factors impeding their attendance, and (3) their suggestions for improvement. Regarding the main staff members, we were particularly interested in (1) their views on the MUKI’s relevance for the female refugees, (2) encountered difficulties, and (3) their suggestions for improvement.

## 2. Materials and Methods

### 2.1. Data Collection and Procedure

The cross-sectional study using semi-structured qualitative interviews took place in the reception and registration center Patrick-Henry-Village (PHV) in Heidelberg-Kirchheim, Baden-Württemberg, Germany. The PHV is a former US barracks converted into a reception and registration center for newly arrived refugees in 2015 [32] (for further information, see [32,33,34,35]). Between February and July 2017, we visited the MUKI and conducted semi-structured qualitative interviews with (1) the female refugees attending the MUKI and (2) the MUKI’s main staff members. Our inclusion criteria were the age of 18 or older and the ability to give informed consent. We did not specify the total number of interviews in advance.

We recruited our interview partners by directly approaching refugee women attending MUKI onsite and asking them whether they were interested in our study. We made several visits to the MUKI during its opening hours to reach as many refugee women as possible within the study period. As for staff members, we focused on the MUKI’s key staff members responsible for and best informed about the MUKI’s services. True to the MUKI’s women-only concept, all staff members were female. Interested refugee women and staff members were informed about the study verbally and in writing. We told them that participation in our study was voluntary and, in the case of refugee women, would have no impact on their asylum procedure. We also informed them about anonymity and pseudonymization procedures, digital recording of the interview data, length of data storage, withdrawal from the study, and data security. Additionally, we told all participants that they did not have to answer a question if they did not wish to.

We contacted a PHV staff interpreter or a telephone interpreter through an interpreter service if the refugee could not converse in German or English. Interviewees were familiar with the PHV interpreter from previous translation occasions. However, the refugee women were still explicitly asked if they felt comfortable with her translating for them. Interpreters working in PHV must sign a confidentiality agreement before starting work at the center. If we used a telephone interpreter, we requested a female interpreter from an interpreter service we have been working with since 2016. The telephone interpreter also first introduced herself, and we then asked the interviewee if she felt comfortable working with her for the interview. Before the interviews, we sent the interview guidelines to the interpreters, and they also signed a confidentiality statement.

As the MUKI is an open plan space, we conducted the interviews in a quiet room located in a building nearby to ensure privacy. First, we collected data on sociodemographic details (age, religion, country of origin, social support, pre-, peri-, and post-migratory distress factors). The sociodemographic questions were available in German and English writing. The interpreter translated the questions for each additional language during the interviews. The interviewer and the interviewee recorded the answers together. Then, the interview was conducted. We approached the key MUKI staff members about our study during their shift. If they were willing to participate, we interviewed them after work. Again, we collected sociodemographic data (area of work, educational level, age, country of origin, history of flight) and then interviewed them. Two female scientific researchers employed at the Heidelberg University Hospital (IT, CDG) carried out recruitment and data collection. One of the scientific researchers has a medical background, and the other has psychological experience. Both were specially trained to conduct the interviews and were supervised by an experienced researcher familiar with qualitative research methods (CN). The interviewee’s name was not mentioned throughout the interview to ensure anonymity.

### 2.2. Semi-Structured Interviews

We conducted semi-structured interviews with (1) the female refugees attending the MUKI and (2) the MUKI’s main staff members. Our research team developed the semi-structured interview guidelines based on the idea of evaluating the MUKI as a low-threshold support offer for newly arrived refugee women and their children, with a particular focus on the attending refugee women’s and staff members’ perspectives. Methodological aspects of Helfferich [36] were followed. The semi-structured interviews, split into target groups (1) and (2), comprised key guiding questions followed by more detailed questions. Appendix A shows the two interview guidelines.

### 2.3. Quantitative and Qualitative Data Analysis

Demographic variables were calculated using descriptive statistics (frequencies, means, and standard deviations) using the SPSS statistics program [37]. The face-to-face interviews were digitally recorded and transcribed verbatim by research assistants using predefined transcription rules. Then, the transcripts were evaluated using the program MAXQDA [38] and analyzed thematically following Mayring’s qualitative content analysis principles [39]. Before examining the textual material, we defined the content-analytical unit of analysis as every statement (single or multiple sentences) referring to one of our key questions. We removed double statements, and double coding was not possible. Then, we went through the transcribed interviews’ textual material and identified a single or few sentences as codes, representing the most elemental unit of meaning [40]. These codes were labeled with a short term or sentence (coding) and summarized into a relevant category. Then, we checked if other codes matched an already defined category or opened a new category. After analyzing 40% of the textual material (after six refugee women interviews/two staff member interviews), we revised the categories and the whole coding system concerning the previously defined categories’ logic. After that, we completed the remaining textual material analysis and grouped the categories into main themes. Finally, the categories and main themes were discussed in detail in the research team and adjusted if necessary [39]. We analyzed the MUKI female refugees and staff interviews separately.

## 3. Results

### 3.1. Sample Description

We conducted semi-structured interviews with 16 female refugees and all five MUKI employees during the study period. Table 1 shows the sample description of female refugees who attended the MUKI and consented to be interviewed (Appendix B provides further sociodemographic details). The interviewed female refugees were between 18 and 64 years old (M = 31.1, SD = 13.32). Of the 16 women, 12 (75.0%) had children and 3 (18.8%) were pregnant. Two females (12.5%) did not complete the basic sociodemographic questionnaire. We interviewed in English with seven (43.75%) women. One woman (6.25%) was able to speak German, while female interpreters (*n* = 2 (12.5%) with a PHV staff interpreter, *n* = 6 (37.5%) with a telephone interpreter) helped us interview eight women (50%). At the time of the interview, the majority of the female refugees had attended the MUKI on more than four occasions (*n* = 9; 56.25%); five women (31.25%) had come to the MUKI between two and four times, and two women (12.50%) had visited the MUKI for the first time. Most of the female refugees had first heard of the MUKI’s services from other refugees in the PHV (*n* = 9; 56.25%). Four women (25%) stated that the “German Caritas Association” (a registered charity) had referred them, while one woman (6.25%) had heard about the service by chance. Two women (12.50%) did not provide information on how they had heard about the MUKI’s service.

Additionally, we interviewed all five MUKI main staff members who were between 18 and 41 years old (M = 31.7, SD = 11.30) and all women. Table 2 shows their sociodemographic characteristics. Four (80%) were German Red Cross employees; one woman specialized in asylum and procedural counseling. One woman (20%) was a qualified midwife volunteering at the MUKI. The employees described providing counselling (*n* = 5; 100%), teaching German (*n* = 1, 20%), language assistance (*n* = 1, 20%), and generally supporting the female refugees (*n* = 4, 80%) as part of their MUKI work.

### 3.2. Results of the Qualitative Interviews with Female Refugees Attending the MUKI

Two hundred forty individual statements were coded; then, they were grouped into ten categories and summarized into four main themes. In the following paragraphs, we will describe the main themes and categories, showing the respective number of codes in parentheses and exemplarily quotations from the interviews. We have marked text passages with * where an interpreter assisted in the interviews.

#### 3.2.1. MUKI Attendance Motives (172)

This central theme included the interviewees’ statements about their respective motives for attending MUKI.

##### The MUKI’s Services (83)

The interviewees greatly appreciated all the MUKI’s services but saw the German language classes as the most important offer. One interviewee stated: T*hey teach you anything; you can learn anything. You can learn even to, to knit. I cannot, I have not learned it before, but I see some other women doing it. And they teach you always how to behave to people, how to talk. And this is better. And we come here to learn the German language, that’s why we are here. And I especially, I love the language. I love to learn the language* (2302AB/23). Many interviewees told us that learning German and getting to know German culture and customs in general was very important to them. One woman told us: Our culture is taken into account. For example, we had had a celebration, and they brought halal food. And I think that’s good because they also take us into consideration. So as a Muslim and as an Arab* (3001FF/12). The interviews also highlighted that the offered childcare was a great relief. They also appreciated that their children could play outside their usual cramped sleeping and living areas and had access to clean toys and a friendly, welcoming environment. One interviewee highlighted: The atmosphere is good and it is a good idea for the children. All day long, they stay in the rooms, little rooms. And they don’t have so many things there. They don’t have a TV or a cuddly toy. That is why, I think it is good. Here, they can play together because they don’t go to school. They don’t go to school, I think so. And they don’t know how to get in contact with other children* (2303BB/21). The interviewed women also cherished being able to reach out to the MUKI staff and felt the MUKI provided them with a stable point of contact.

##### Socializing with Other Female Refugees (58)

Contact with peers was cited as a significant motive for attending MUKI. They felt that they were able to exchange experiences, reduce prejudices, and create mutual distractions there. One woman pointed out: That there are so many people from different countries, this made me stronger; if I met different cultures or different nationalities, I would have said, in the beginning, that I don’t know how they think or behave. So, outside, I now can start talking; that is not a problem for me anymore because I made the experience in here that all are nice* (3002FF/14). Furthermore, interviewees described feeling a sense of community. An interviewee answered: It is calming and relaxing to get outside and let the kids play with each other. You have somebody who listens to you in an atmosphere without pressure. For sure, topics like asylum application arise, and we give each other help* (2304CC/37). Above all, they underlined that the MUKI facilitated cultural exchange, acceptance of different religions, and cultural backgrounds and fostered respectful interactions. One refugee woman said: *All, yes, all, with all nationality. There is something amazing to meet people from other countries. To see sometimes talking about traditions, sometimes talking about what they are thinking about. It’s beautiful. I like them all* (1954AA/26).

##### Friendly Sheltered Atmosphere (31)

Interviewees valued the MUKI as a friendly environment and felt that the MUKI’s welcoming atmosphere contrasted strongly to their sparsely furnished accommodations. One woman described: *Everything is just relaxed. Everybody is looking happy. So, another world, I think. [..] For, for a moment, I forgot totally I’m in the camp* (1711FN/57). They said that they felt more relaxed and calmer when they attended the MUKI and that the sheltered space fostered a sense of security. One interviewed woman noted that she was able to find “inner peace” there. Another woman explained: I am so, so happy about the MUKI. There, I can relax my soul. If it did not exist, I would have gotten crazy* (3002FF/18).

#### 3.2.2. Factors Impeding Attendance (12)

The interviewed female refugees named intrapersonal and interpersonal difficulties in attending the MUKI.

##### Intrapersonal Factors (5)

A few interviewed women said they felt overwhelmed during their first visit because no one had introduced them to the MUKI services and premises. *I did not know where to start. Yes, I think that’s one thing; I don’t know how they can fix. [..]. So, I was just lost* (1711FN/29). Other women reported that the MUKI’s services could only provide short-lived distraction and comfort from their troubles. One interviewee stated: During the time I attend the MUKI, I forget about my problems. But as soon as I leave, all the problems come back, for instance, about my sick son and that she is threatened by deportation to another country* (2304LL/21). Some pointed out that they sometimes had difficulties finding the energy to come after not sleeping because their children had cried all night.

##### Interpersonal Factors (7)

Some women reported that new female refugees were sometimes deterred by the noise and presence of so many women in the MUKI’s limited space. One female said: *I would put the children in here or another room, separated room because I want to and I like to concentrate on the teacher without hearing noise* (1954AA/55). Furthermore, one woman stated that she had not felt included by the other women during her first visit and found it challenging to engage with them. She said: *The first time I came, it was not very easy because I was alone, nobody to talk to. Everybody really is talking* (1711FN/25). In addition, she added that children of all age groups played together in the same room due to the limited available space, which could sometimes feel too crowded and noisy for some women looking for a retreat.

#### 3.2.3. Suggestions for Improvement (25)

In the interview, the female refugees made several suggestions on how the MUKI’s services could be improved, including offering more services and fostering the women’s sense of community.

##### Expansion of the MUKI’s Services (18)

Most of the interviewed female refugees would like to see an expansion of the MUKI’s services. They especially asked for more, ideally daily, German classes. One refugee woman explained: *If they learn us if they would give us another two hours of learning, I would be really happy. They give a learning from 10 to 12. Very intensive information, very useful. Another two hours. I like learning* (1954AA//75). They also hoped for additional German lessons for children, more experienced German teachers, and more interactive lessons. They also expressed their desire for more leisure and education materials, particularly CDs, films, books, and musical instruments. *I don’t know if they have, maybe, books. Because someone like me, I like poetry. As maybe some books to read. Maybe in English* (1711FN/87). One woman specifically hoped for longer MUKI opening hours. Some of the women desired a separate children’s playroom and more support offers for their children. One interviewee pointed out: There are some children who are aggressive. They come from war regions, and what did they see? Only war and blood. I think it would be better if a woman could help them* (2303BB/34).

##### Strengthening the Sense of Community (7)

The women made specific statements concerning possible ways to improve the MUKI women’s sense of community. They suggested that their cohesion might increase through regular joint activities, games, or simply tidying up together at the end of the day. One woman said: *Maybe they organize some games, people play. Not just toys. We can organize small competitions, small presentations. What do you think? Because I check somewhere at another office and so I saw the right conferences and stuff. We can make our little presentations here* (1711FN/92). She further stated that women might enjoy holding short, informative presentations on various topics, such as their cultural or culinary background, for each other.

#### 3.2.4. MUKI as a Women-Only Space (31)

Most female refugees welcomed the MUKI’s male-free concept, but it also led to some concerns.

##### Facilitating Open Engagement (16)

Most women supported the MUKI’s women-only concept and reported being able to engage more freely and open-mindedly with each other in a safe, male-free environment. An interviewee answered: *We can do everything that we want. If we were men here, we cannot do it, I think. To speak about everything, to talk, to do something. Because I am, for myself, I am afraid about other people. I am a closed woman. It is better that it’s only women* (2401AA/55). One woman said she felt it was sheltered enough for her to take off her headscarf, while another noted that she could breastfeed openly at the MUKI without being censored or sexualized. One interviewee stated: I think it is a very good atmosphere because you come together and for a short time, you can leave your problems behind. I personally do not have any problem with men. But I think it is easier if there are no men because women with each other can have fun, can laugh, and can share their problems. If men would be around, the females would be shier than now* (2304LL/19).

##### Feeling Safe (8)

Many women reported feeling sheltered and secure in the MUKI’s male-free environment. One woman said that she feared men in general, while two other women stated that they felt nervous and insecure in the presence of men. One commented: I think that’s good because then I can move around here easily. And also sit down in a very relaxed way* (2307EE/14). Furthermore, the women remarked to experience interpersonal difficulties with men generally. One interviewee mentioned that during the German class: *If men are there, I don’t ask anything because I am nervous. This house is free. All are women, no nervousness, and free talk* (1801AM/47).

##### Concerns (7)

The MUKI women-only concept also led to some concerns. The women said that male-female segregation was not common in Germany and felt that it might help them interact with men in a mixed environment. A refugee woman pointed out: For me, the most important is the German class. Therefore, for me, it does not matter if men are around who want to learn the language as well because, without language, you don’t have a future. It would be mixed, as in school or university, that’s why I would not mind if men were present* (2305DD/51). Some women also felt that a similar program for men would benefit male refugees to experience community and support. On statement was: Men should have the same like this, only for men. And they must have been told, for instance, that their wives have to have contact with men while going to school, studying something. They have to understand that in Germany it is different. Yes, it is so important that men understand this* (2303BB/27).

### 3.3. Results of the Qualitative Interviews with MUKI Staff

We coded one hundred sixty-seven individual statements and then grouped them into seven categories and subsequently summarized these into three main themes. We again describe the main themes and categories in the following paragraphs illustrated with quotations translated from German to English. The respective number of codes is shown in parentheses.

#### 3.3.1. The MUKI’s Relevant Features (93)

The staff identified key MUKI features that they believed made attending the MUKI project particularly attractive to the refugee women.

##### The MUKI’s Atmosphere (30)

The MUKI staff explained that the MUKI exerted a calming and relaxing effect on the women. One staff member pointed out: ‘I think the atmosphere is important. Everybody is welcome. Everybody can talk to each other. Sometimes there is a German language course. But even if there is no German language course, there is still a lot going on here. They feel very comfortable here. It’s a change from staying in their room, a change of scenery’ (2002CR/53). They highlighted how they could see that the women benefited from the MUKI’s friendly atmosphere in which they experienced a sense of acceptance and inclusion. Furthermore, one staff member stated: ‘I had trauma training. And there, we were told that it is essential for individuals with forced migration experiences to maintain their independence and feel that they have something under their control. I think that this can be encouraged here with the German classes and crafting. The women feel less passive but can be active. I think this also helps them with their integration process if they can learn some German and engage with others’ (0506CS/51). Interviewed staff members emphasized that the MUKI’s women-only policy helped the refugee women feel comfortable and more at home there. One staff member said that she felt that the women were invigorated after attending the MUKI: ‘Here, the atmosphere is open and relaxed. The women can be themselves, can contribute. They don’t have to ask or come as petitioner; we do everything together here’ (0506CS/59).

##### The MUKI’s Services (36)

The staff felt that the MUKI’s services provided the women with somewhere to go and something to do. One interviewee stated: ‘Every Friday, there is an offer where a lady brings many very nice craft supplies and crafts and paints with the refugee women. This keeps them busy and takes their mind off things. We always have sheets and pens here which the women and children can use. And books and booklets they can read. Especially, children’s books need to be on the bookshelf. Once, a woman asked me if she can read to me to practice her German’ (0407ZW/33). The staff members felt that the MUKI provided the refugee women with distraction from their everyday worries, problems, and burdening thoughts, while the services also improved their sense of self-efficacy and control despite their difficult situation. One interviewee stated: ‘Yesterday, there was a celebration and the place was packed. I think there were more than 35 women here. This was a real highlight because they can enjoy eating something else, too. And it is something special; you can forget everything. They danced to the music; there was this feeling that no problems existed here—it was as if they were back home, in their country’ (0506CS/38). The staff felt that the MUKI enabled the women to practice their “soft” skills, including openness and sociability, after experiencing much hostility during their flight. Additionally, the staff pointed out that the MUKI’s fixed opening hours helped maintain a daily routine. The provided childcare services also enabled the women to recuperate and engage: ‘The women don’t have to leave their children somewhere. They can take them along. I think that this is very important for the women’ (2002CR/63).

##### Socializing with Other Female Refugees (27)

The staff felt that the interaction and sense of community between the refugee women across all cultural and religious backgrounds was an essential experience for the women: *I think they are more confident in engaging with other women. And that they have friends to meet with—it is an important development that they understand that they can approach others and talk to others. That they can approach us and create the first contact with Germans and with other women. That is so nourishing* (0407ZW/51). They described how if the refugees did not speak the same language, they would exchange the information nonverbally. They also mentioned women supporting each other in learning German or showing each other different headscarf tying techniques. One statement was: *They teach each other German. This is impressive to see. It does not matter from which country they are; if one is better than the other, she teaches the other. That is amazing. And it really goes across cultural barriers* (0407ZW/33).

#### 3.3.2. Factors Impeding Work at the MUKI (52)

This central theme summarizes staff statements regarding experienced difficulties while working at MUKI.

##### Individual Difficulties (13)

The staff described their work at MUKI as stressful and exhausting. Many felt very responsible for the refugee women and found it challenging to keep their professional distance, sometimes feeling too emotionally entangled with their stories. One staff interviewee mentioned: *It is tough hearing the stories. For instance, if you hear that they have not seen their families for five months and that they are very sad about that, it really hurts me. You have to watch out for yourself. And that’s the attitude I come here with because, I know, that it is very close to my heart and I have to be careful* (0407ZW/61). *I have been working with refugees for a year now. In the beginning, it was tough for me to keep some professional distance. But now, I think, I have to take care of myself because if I don’t, then I won’t be able to do a good job anymore* (2002CR/155). The MUKI’s counseling services, which include asylum and procedural counseling, were so popular that the staff reported often working nonstop to ensure that all women received the help they needed. One interviewee stated: *It would be so nice to create more projects for the women. […] But you just can’t manage it when you are working here and have to take care of everything: little activities, little pleasures. I would really like to include more, but it is hard. We are already overburdened as it is* (0407ZW). Staff also reported that it was difficult for them to keep track of so many refugee women attending MUKI.

##### Interactional Difficulties with the Refugee Women (12)

The MUKI staff described having trouble engaging with some attendees, especially when they perceived the women as very demanding and impatient toward them. One statement was: *I have the feeling that since the expedite asylum procedure started, the individuals are more demanding, impatient, and sometimes more aggressive* (2002CR/125). One staff member said: *In the beginning, I was always very cautious with touching the refugee women. And I always asked—I still do this. And then, there was this one lady who really shouted at me: You are the doctor. Just do your job and don’t ask me all the time* (2709BTr/65). They also reported that they had to deal with territorial behavior among the women. On one occasion, a group of refugee women had attempted to take over the MUKI, preventing other potential MUKI attendees from coming in. For instance, one staff member told us: *Once, there was a strong group of women. They were a quite dominant unit. […]. I defended another woman who the women wanted to send away because she had been too loud with their children. The group of women wanted to create a calm place and place to sleep. And the woman with her children was attacked. […] And of course, I stood by this one woman, and then it really escalated* (2002CR/99).

##### General Conditions (27)

The staff remarked that available information regarding the PHV’s psychosocial services was not disseminated enough and thought it was unfortunate that not all women were informed about the MUKI’s services. Furthermore, they critically noted that turnover was high in both refugee women and MUKI staff. One staff member stated: *When the women leave, they leave with positive experiences. Others may not yet know exactly what MUKI is. To advertise the MUKI, we offer group meals or parties. Some women have been transferred again. So, we have lost a few experienced women again. Sometimes that’s a bit of a shame, but that’s the way it is* (0506CS/67). In addition, they pointed out that the children made the MUKI a noisy environment to work and be in. One staff member added the following regarding the female-only environment: *I am conflicted somehow. There are great facilities for women, but I also see different offers for refugee females and unaccompanied refugee minors, and the men are always forgotten. […]. I think that the slogan ‘this is only for women’ sends out the wrong message—as if all refugee men were criminals* (2002CR/171).

#### 3.3.3. Suggestions for Improvement (22)

The employees made several suggestions for improvement, which were mainly related to conceptual aspects. All staff members advocated extending the MUKI’s opening hours, holding more German lessons, and expanding the MUKI’s services in general. One woman pointed out: *We hope we will get a few more co-workers soon so we can open again in the afternoon. We could then have activities in the mornings and offer quiet recreational times in the afternoons* (0506CS/65). To address the MUKI’s noise level, the employees suggested introducing rest periods during the opening hours. Generally, the staff said they would like to see a broader range of offers for women and men outside MUKI. One woman stated: *Of course, the optimum would be more German classes. But then, quiet days would be unthinkable inside the MUKI. I think that the women would also appreciate it if they could do something else here, too. And of course, extend the opening hours, but that is not possible right now because of low staff resources. I also think it would be good if we had a kind of terrace where you could sit outside together in summer* (2002CR/67).

## 4. Discussion

This study aimed to evaluate a mother–child center (MUKI) providing psychosocial support to newly arrived female refugees in a registration and reception center from the attending female refugees’ and MUKI staff members’ perspectives. Our results suggest that the MUKI has the potential to establish itself as an important women’s hub in the reception and registration center. The interviewed female refugees appreciated the MUKI’s psychosocial services and sheltered environment. The interviewees generally reported few attendance barriers, which were primarily related to intra- and inter-individual issues. The staff mainly reported difficulties regarding the MUKI’s general working conditions, including insufficient awareness of the provided services and a high turnover of staff and attendees. Interviewees and staff alike were in favor of expanding the MUKI’s program.

The refugee women’s statements showed that the MUKI’s diverse offer, the exchange with others and the welcoming atmosphere motivated them to attend. The interviewees particularly valued the German classes. Deacon and Sullivan [18] highlight that host country language skills can facilitate refugees’ successful adjustment. However, as refugee women often have less formal education and weaker foreign language skills, they tend to have more adjustment difficulties. In their study, refugee women stated that inadequate language skills hindered them from forming social networks, reaching out for help, and accessing essential resources [18]. Teaching refugee women language skills and providing low-threshold services can promote their community participation and help them develop agency and empowerment. The interviewees’ comments show that MUKI fostered the women’s self-efficacy on an inter- and intra-individual level: the women frequently voiced feelings of social acceptance and mutual respect and a sense of security safety in connection with their experiences of the MUKI. Considering the women’s harrowing gender-specific reasons for and experiences during flight [3,4,5,6], creating a setting such as this is a first step in providing women with the necessary foundation for stabilization and emotional distancing from an experienced trauma. Women’s programs are critical. They offer at-risk groups a safe starting point for healing without coercion to self-disclose or shame. The experience of positive emotions and community are protective and stabilizing factors for refugee women [41,42]. Moreover, positive social interaction and acceptance are crucial mental health resources and are known coping strategies for dealing with challenges and acculturation difficulties [43,44]. Across cultures and populations, a sense of community has been identified as an essential resource promoting a collective sense of coherence that supports mental well-being and life adjustment [45].

Although the interviewed female refugees experienced the MUKI as a low-threshold support offer, overexertion, social engagement-related difficulties or fears, and its generally high noise level were seen as key attendance barriers. Intrapersonal factors, particularly anxiety, impede care access [46], and newly arrived refugees are often unfamiliar with the host country’s support services. Some of our interviewees stated that they felt overwhelmed when they first attend the MUKI. A peer or staff mentoring program could help take down this barrier. Designating an experienced staff member or long-time attendee as a point of contact may make it easier for women during their first visits to the MUKI. Mentors could make new attendees feel more welcome by showing them around and explaining the available offers. A welcoming ritual (e.g., a weekly greeting session) might also help women get to know each other and socialize more quickly despite the high turnover.

All the interviewees felt that the MUKI’s noisy environment was a deterring factor. They suggested the introduction of daily rest times to address this issue. Unfortunately, the MUKI’s high noise level is difficult to control because its large, open-plan layout is used by many people for different purposes simultaneously. In light of the interviewees’ appreciation and need for the service, it would be great to expand the MUKI’s premises and offers with sufficient funding. However, the PHV is located in former military barracks, which were not built to accommodate refugees. The refugees’ difficult living situations are well documented: the accommodation’s general condition is poor, the noise level is high, and privacy is hard to find in the shared rooms and showers [47,48]. Furthermore, the structural barriers to medical and psychosocial services are also known [49].

While the women appreciated the MUKI as a safe space, they highlighted that it could not help them with their severe long-term concerns. Post-migratory stressors and their effects on refugees’ psychological stability, especially refugee women, have been demonstrated in previous studies [14,50]. The MUKI is a low-dose, low-threshold support offer providing refugee women with a safe space to socialize, advance their skills, and maintain a daily routine (e.g., German language or knitting classes). It also offers midwifery assistance and asylum counseling to address some post-migratory needs. At the very least, it gives the refugee women some distraction from their otherwise very monotonous days at the PHV and long-term worries. However, it cannot change post-migratory stressors, such as residency, family reunification, or provide trauma therapy.

The interviewed women emphasized that they had benefited from the MUKI women-only space. It had enabled them to interact more openly and experience feelings of freedom, ease, and safety. The women saw the MUKI as a space for personal growth and healing after their traumatic flight and personal history. Our results corroborate previous calls for more gender-sensitive services and gender-specific access to care [4,6]. Nevertheless, Kraus [30] warns against women’s stigmatization as victims and assigning them a per se vulnerable position and inferior social status. She pointed out that refugee women’s empowerment in social contexts requires both men and women to renegotiate and redefine their roles, identities, and relations [30].

Interestingly, some of the interviewed female refugees expressed reservations about the MUKI’s male-free concept. While safe spaces are necessary and valuable to increasing well-being and addressing gender-specific needs, the idea systematically excludes men. Some interviewed women noted that a male-free space was unfair toward refugee men, potentially stigmatizing, and unrepresentative German culture. This may be true; however, refugee women are still at risk for gender-specific violence despite resettlement. Bartolomei et al. [51] showed that resettled refugee females in Australia faced discrimination for being single or having an illegitimate child, forced marriage, domestic violence, and engagement in survival sex. The need for gender-sensitive shelters and psychosocial services for newly arrived pregnant and refugee women, such as the MUKI, is great. Despite limited funding, it would be good to have a similar program for men in the PHV addressing gender issues and promoting equality between female and male refugees. Organized cross-gender and nationality community meetings could improve morale and well-being as well as relieve tensions.

The refugee women had specific ideas about what they wanted to change concerning the MUKI concept. They wanted to see an increase in the number of courses, the provision of more materials, and, more specifically, more joint activities to strengthen their sense of community. The women’s ideas reflect their high regard for the MUKI. Olivius [52] stated that the refugees’ participation in refugee camps could improve protection and assistance and foster self-reliance. For the refugee women, giving us feedback as quasi-representatives of their host country could also have a participatory aspect in the sense of “being heard” or “having a voice”. Kreitzer [53] asked refugee women about their perceived barriers in engaging in program planning in refugee camps. The assessed women frequently gave the lack of childcare as their main reason for non-engagement. In our study, the women asked for more courses and materials specifically for children and, above all, for more support for children in need. Although children are have been recognized as highly vulnerable group among refugees and child-oriented services are extremely sought after, current literature has documented a great lack of offers to date [54]. Bronstein et al. [55] summarize that refugee children experience high psychological distress, with prevalence rates for PTSD ranged from 19 to 54% and depression ranging from 3 to 30% in their review. Internalizing and externalizing problems were frequently found [55]. In refugee camps, adequate toys or other child resources are often sparse. Refugee children must often shoulder responsibility early on, are very closely attached to their parents, and often have limited peer contact. From a psycho-developmental perspective, providing refugee minors with a safe, clean, and pleasant environment to be and play as the children is an essential step on their long road to healing [55].

Regarding the relevant factors motivating MUKI attendance, interviewed MUKI staff members’ statements were consistent with the refugee women’s descriptions. This suggests that the staff members could convey the MUKI’s gender-sensitive and culturally adapted offers and conceptual relevance and that the refugees could appreciate this. Therefore, the staff members need to have a culturally sensitive attitude and communication skills. According to Brooks et al. [56], cultural sensitivity ‘requires an awareness of cultural diversity, including how culture may influence patients’ values, beliefs, and attitudes, and involves acknowledging and respecting individual differences’ (page 384). Recently, different health researchers have focused much attention on evaluating and improving cultural sensitivity in communication, including healthcare in general [56], outpatient psychotherapy [57,58], and nursing care [59].

The staff members mentioned few concerns regarding the women-only concept. Yet, the MUKI’s safe space for women can also be seen as disadvantageous: Women and men forfeit the chance to socialize in a sheltered, supervised environment preparing them for German culture. However, male-free areas, such as women-only sports, educational programs, and (international) women’s cafés, are also common in Germany. The MUKI’s concept aims to strengthen refugee women’s resources shortly after they arrive in the host country. Nesterko and Glaesmer [60] note that it is almost impossible for newly arrived refugees and refugees waiting for a decision on their asylum procedure to fully structurally and internally ‘arrive’ in their host country in light of the many uncertainties. They also point out the refugees’ rupture in their relationship with their home country and thus identity. A successful acculturation process always includes participation in both the country of origin and the host country’s culture [60]. Understandably, the greater the cultural distance between the host and home country, the more significant the perceived migratory burden [58]. In this respect, you could say that the MUKI provided the interviewees with a feeling of familiarity and offered them an interim cultural ‘home’. The MUKI’s protected environment allows women from more restrictive, gender-segregated countries in particular to enjoy more freedom and open interaction.

Concerning work-related difficulties, the MUKI’s staff members described their work as strenuous and stressful. They emphasized their high workload, the high turnover of staff and attendees, and the female refuges’ great need for support. Many employees reported a strong sense of responsibility for the refugee women. At times, they felt emotionality enmeshed and had difficulties in keeping their professional distance. The concept of secondary traumatic stress and burn-out has gained importance in literature and clinical care in recent years. The Diagnostic and Statistical Manual of Mental Disorders Fifth Edition (DSM-5 [61]) has recognized secondary traumatic stress’s impact. Depending on the profession, the prevalence rates for secondary traumatic stress and burn-out are heterogeneous: While PTSD (17.1%) and burn-out (57%) symptoms were high in Greek rescue workers [62], nurses working in a regional trauma center in South Korea showed levels of secondary stress symptoms around 84.4% [63]. In the United States, up to 30% of refugee caregivers exhibited high secondary traumatic stress levels [64]. In contrast, medical students volunteering in a reception center for refugees (3.2%) [65] and humanitarian aid workers in Jordan report low secondary traumatic stress levels (4% burn-out, 7% secondary traumatic stress) [66]. Similarly, Akinsulure-Smith et al. [67] pointed out that passive coping strategies, including general distraction, venting, substance use, behavioral disengagement, or self-blame, were associated to higher secondary traumatic stress and burn-out risk. In contrast, self-efficacy [68], personal commitment, organizational support [69], emotional intelligence, use of active coping, emotional coping, and positive reframing [67] are established protective factors. Wirth et al. [70] uncovered the high workload and caseload of social workers working with refugees and homeless people and recommended increasing the number of professionals and decreasing their caseloads. The authors point out that frustration increased when the employee felt unable to care for clients adequately [70]. As most interview statements referred to difficulties related to the general working conditions, more efforts are needed to improve the MUKI’s employees’ working conditions. The employees’ suggestions, such as extending opening hours, employing more staff, and introducing set rest periods, may help address the noise level and high workload. Isawi and Post [68] highlighted the need for supervision, training, and self-care practice, such as meditation, mindfulness, physical exercise, and social support in taxing working environments.

## 5. Implications and Future Directions

Our results suggest several implications and future directions: The MUKI offers a low-threshold, low-dose, women-only environment in which female refugees’ gender-specific needs can be addressed. It has great potential in further establishing itself as an information hub and psychoeducation center facilitating cultural exchange and increasing self-empowerment. Women-specific offers have been shown to improve access to care structures and increase women refugees’ integration and participation. Policymakers and relief organizations responsible for registration and reception centers should consider implementing nationwide MUKI programs for refugee women and children. As discussed above, there are projects worldwide addressing refugee women’s needs. Nevertheless, more (shelter) programs are needed for refugee women who have faced/are facing trafficking, gender-based violence, and harassment. In cooperation with the German Interior Ministry, programs are currently being developed to identify trafficked women via screening tools and promote referral to counseling services and therapist, social worker, and lawyer networks.

Moving forward, refugee women attending the MUKI should be more involved in planning and implementing activities: First, the MUKI’s opening hours should be extended. This would allow the women to creatively organize their time and ideas, such as talking about cultural issues and showcasing skills. Second, additional group offers or competitive games should be introduced. This could strengthen the overall sense of community, encourage self-empowerment, and foster intercultural exchange. Third, more experienced MUKI attendees (frequent attenders) should have the opportunity to take on more responsibilities in the MUKI and develop their sense of self-efficacy. Attendees should be encouraged to raise awareness for the MUKI’s services and to mentor new attendees. Resident refugees can apply for work placements in the PHV, which include working in the gym, serving food, or keeping the premises clean. Consideration should be given to whether such a position could also be created for the MUKI. Fourth, more psychoeducational offers (e.g., lectures, workshops) covering German culture, (mental) health literacy, contraception, communication skills, women’s and girls’ rights, and equality should be introduced. This could lead to a better understanding of life in Germany and help prevent gender-based violence and provide early access to (mental) health care if needed. In addition to psychoeducational sessions, weekly focus groups could strengthen the sense of community among the attending refugee women. It would be interesting to follow-up if the focus groups could also continue online after redistribution to different shelters to build more lasting relationships between the women.

The interviewed staff members also advocated expanding the MUKI offers and opening hours, and introducing rest periods. Given the heavy workload of MUKI staff, serious consideration should be given to including or employing refugee women as staff in a supporting role. That said, the MUKI still requires qualified and long-term employees to shoulder the workload and opening hours. More funding is needed for MUKI to improve working conditions and implement new conceptual ideas.

On an organizational level, it may help if staff members and interested refugee women received workshops to reinforce culturally sensitive communication and self-care workshops, such as mindfulness and meditation training. In addition, psychoeducation on secondary traumatic stress, burn-out, and compassion fatigue should be addressed. To our knowledge, MUKI staff members already receive regular supervision. However, considering their work with highly traumatized individuals, further staff support offers should be established. Challenges, such as language barriers and rapid transferal to other locations, must not be overlooked.

Future qualitative and quantitative research should examine self-empowerment, sense of (community) coherence, and effects of psychoeducation among refugee women attending MUKI more closely. It would also be interesting to investigate secondary traumatic stress, culturally sensitive communication skills, work-related self-efficacy, and MUKI staff profiles.

## 6. Limitations

This qualitative study has limitations that need to be addressed: Firstly, we cannot rule out social desirability-related response biases during the interviews. Secondly, we only assessed the perspective of refugee women who had attended the MUKI. Women who might have considered attending but did not were not interviewed. Unfortunately, we did not record how many refugee women declined participation and why. We estimate that approximately 70% of the women we approached participated in our study, but we cannot specify a drop-out rate. In the case of MUKI staff, we focused on the key employees. We felt that their intimate experience of the service would provide comprehensive insight. We did not include temporary MUKI workers in the study. Third, the number of interviewees in our study is small (16 refugee women and five staff members). We did not pre-set the number of MUKI interviews for the following reasons: (a) this was the first study we conducted there; accordingly, we did not know how many refugee women would be willing to participate in our survey at the outset; (b) the MUKI is a drop-in center open to all refugee women and their children. Rapid reassignments to other shelters leads to fluctuating attendance and poor predictability are characteristic of all PHV-based services. That said, qualitative research aims to capture the participants’ perspectives by systematically analyzing the collected narratives. Often, the concept of data saturation, referring to the point when no new information is discovered in data analysis in the research process, is used to provide insight into the quality of the categories and major themes identified during the analysis. While different recommendations regarding the sample size of interviews exist (range from six to 50 interviews), Hagaman and Wutich [71] note that saturation of the most common themes should be reached within 16 interviews. In relatively homogeneous groups on focused topics, 13–31 interviews might be required to identify significant themes. Following these recommendations, our study’s sample size of refugee women attending the MUKI was sufficient to achieve data saturation. Furthermore, we conducted five interviews with the key MUKI staff members. Although the total number of staff member interviews is small, we interviewed all primary employees. Nevertheless, the interviews’ validity may be limited by the small sample sizes in both groups. We cannot rule out a biased representation of the MUKI’s acceptance. Fourth, the interviews were conducted in 2017. However, the MUKI concept described in this study is still up to date. Before the COVID pandemic, no fundamental changes were made to the services provided by MUKI. Since the onset of COVID in early 2020, MUKI services have had to be significantly reduced, and participation has been curtailed in line with severe public and private life restrictions implemented nationwide. The confined living conditions are highly conducive to coronavirus infection in the shelter. Although urgently needed, all projects operating within the center can only be carried out with restraint, under great caution, and in compliance with the regulations and hygiene standards in force. However, especially in times of COVID, safe spaces for refugee women and their children remain crucial. Fifth, due to the setting inherent high turnover of refugee women in the PHV reception and registration center, no attention was paid to heterogeneous distributions of age, education level, and country of origin when selecting interview partners. While we focused on the experience of the MUKI as a low-threshold psychosocial service for pregnant refugees and refugees with children, we did not assess possible cultural differences, which might have affected the responses of the refugee interviewees. However, we did not know which refugee women would attend the MUKI and participate in the study and did not select the interviewees according to their cultural background

## 7. Conclusions

Female refugees are frequently exposed to gender-specific dangers before, during, and after their flight while often carrying the sole responsibility for their accompanying children. Their specific care needs are rarely addressed in their destination or host country shelters. Our data suggests that the MUKI has established itself as an important hub for newly arrived female refugees and their children by providing psychosocial care and a sheltered environment. Our findings show that refugee women appreciated the MUKI’s offers, including German classes, childcare, leisure activities, and socializing in a welcoming atmosphere. The majority of the refugee women valued the MUKI’s women-only concept enabling them to feel free and safe. The interviewed refugee women were also eager to participate in the planning and implementation of future MUKI offers. Data have shown that addressing gender-specific needs can help refugee women in their empowerment, self-efficacy, and personal growth.

In the future, the MUKI needs to address attendance barriers and take the attendees’ ideas into account. Establishing a mentoring or buddy system (refugee to refugee) and expanding MUKI opening hours could be critical next steps. Policy efforts to introduce the MUKI concept as a gender-specific care program for vulnerable refugee groups in registration and reception centers at a national level are ongoing in Germany. Future services should include psychoeducation and information on culture, (mental) health literacy, contraception, and women’s rights to promote gender equality and improve future integration. The refugee women need to be actively involved in the program development and implementation.

Furthermore, long-term screening and protection programs for refugee women affected by trafficking, gender-based violence, and harassment are desperately needed. A multidisciplinary approach involving therapists, social workers, and advocates should be adopted and evaluated for feasibility and effectiveness. Furthermore, more attention needs to be paid to refugee aid workers working conditions. More studies assessing work-related mental health problems, such as secondary traumatization, are required. Refugee aid workers’ coping strategies and intercultural skills should also be examined. Future research must improve the identification of female refugees affected by gender-based violence and trafficking and develop interventions to improve literacy, psychoeducation, and overall empowerment of refugee women. These efforts must not end in registration and reception centers but should also extend to subsequent shelters and municipal housing.

## Figures and Tables

**Table 1:** Sample description of interviewed female refugees attending the MUKI (*N* = 16).

Female Refugees Attending the MUKI (*N* = 16)		
Region of Origin ^a^	*n*	%
Sub-Saharan Africa	3	28.8%
Middle East	6	37.6%
South Asia	3	28.8%
South-Eastern Europe	2	12.5%
Not specified	2	12.5%
Religion		
Christian	6	37.5%
Islamic	4	25.0%
Not specified	6	37.5%
Social support ^b^		
None	5	31.3%
Partner/husband	6	37.5%
Children	8	50.0%
Parents/siblings	4	25.0%
Friends	2	12.5%

^a^ = Sub-Saharan Africa includes interviewed refugees from Cameroon and Eritrea; the category Middle East includes interviewed women from Syria, Iran, and Palestine; the category South Asia includes interviewed women from Sir Lanka and Pakistan; the category South-East Europe includes interviewed women from Albania and Macedonia. ^b^ = Multiple answers were possible.

**Table 2:** Sociodemographic sample description of the MUKI staff (*N* = 5).

MUKI Main Staff Members (*N* = 5)	*n*	*%*
Home country		
Germany	4	80.0%
Iran	1	20.0%
History of flight		
No	5	100%
Yes	0	0.0%
Educational level		
Abitur ^a^	1	20.0%
University degree	4	80.0%

^a^ = In Germany, the Abitur is a valid school certificate for admission to any university-level study or vocational training program.

## Data Availability

The datasets used and analyzed during the current study are available from the corresponding author on reasonable request.

## Appendix A

**Table A1:** Semi-structured interview guidelines for the interviews with female refugees who attended MUKI (N = 16) and with the MUKI’s main staff members (*N* = 5).

Interview Guide for the Interviews with Female Refugees Attending the MUKI
Why do you attend the MUKI?
How do you like the MUKI?
How do you like the atmosphere?
What positive effects does it have on you?
What suggestions do you have for improving the MUKI?
Interview guide for the interviews with the MUKI staff
What do you think is important for the refugee women attending the MUKI?
What difficulties do you experience in your work at the MUKI?
How could the MUKI be improved?

Note. MUKI = The mother–child center is located in the state registration and reception center for refugees ‘Patrick-Henry-Village’ in Heidelberg-Kirchheim, Germany.

## Appendix B

**Table A2:** Further sociodemographic data of interviewed female refugees attending the MUKI (*N* = 16).

Female Refugees Attending the MUKI (*N* = 16)		
Reasons for flight		
Loss or threat to the family	6	37.6%
Domestic violence, abuse, rape	4	25.0%
Witnessing a homicide	2	12.5%
War	2	12.5%
Lack of medical care	2	12.5%
Lack of economic prospects	2	12.5%
Family reunion	2	12.5%
Discrimination	2	12.5%
Political persecution	2	12.5%
Not specified	2	12.5%
Flight-related burdens		
Lack of food, hunger	2	12.5%
Duration of flight	3	18.8%
Threat to their lives	6	37.5%
Death of family members/relatives	1	6.3%
Abuse, rape, experience of violence	3	18.8%
Illegality	2	12.5%
Separation from the family	1	6.3%
Accommodation-related burdens		
Refusal of the asylum application	2	12.5%
Noise, agitation, lack of privacy	10	62.5%
Hygienic deficiencies	4	25.0%
Discrimination	2	12.5%
Fear of violence	1	6.3%
Physical assaults	2	12.5%

MUKI = Mother–child center within the reception and registration center “Patrick-Henry Village” in Heidelberg-Kirchheim, Germany. Multiple answers were possible.

## References

[1] United Nations High Commissioner for Refugees Global Trends. Forced Displacement in 2018. https://www.unhcr.org/statistics/unhcrstats/5d08d7ee7/unhcr-global-trends-2018.html.

[2] Nikendei C., Greinacher A., Sack M., Borcsa M., Nikendei C. (2017). Traumafolgestörungen und psychische Komorbidität: Konzeption und Diagnostik. Psychotherapie Nach Flucht und Vertreibung [Psychotherapy after Flight and Expulsion].

[3] Yazid S., Natania A.L. (2017). Women Refugees: An Imbalance of Protecting and Being Protected. J. Hum. Secur..

[4] Schouler-Ocak M., Kurmeyer C. Study on Female Refugees. Repräsentative Untersuchung von geflüchteten Frauen in Unterschiedlichen Bundesländern in Deutschland. https://female-refugee-study.charite.de/fileadmin/user_upload/microsites/sonstige/mentoring/Abschlussbericht_Final_-1.pdf.

[5] Sansonetti S. (2016). Female Refugees and Asylum Seekers: The Issue of Integration.

[6] Freedman J. (2016). Sexual and gender-based violence against refugee women: A hidden aspect of the refugee “crisis”. Reprod. Health Matters.

[7] Baye E.M.-O., Heumann S. (2017). Migration, Sex Work and Exploitative Labor Conditions: Experiences of Nigerian Women in the Sex Industry in Turin, Italy, and Counter-Trafficking Measures. Gender Technol. Dev..

[8] United Nations High Commissioner for Refugees Action for the Rights of Children (ARC): Critical Issues—Abuse and Exploitation. https://www.unhcr.org/protection/children/3bb81aea4/action-rights-children-arc-critical-issues-abuse-exploitation.html?query=exploitation.

[9] Bundesamt für Migration und Flüchtlinge (2020). Das Bundesamt in Zahlen 2019.

[10] Müller U., Schöttle M. (2005). Lebenssituation, Sicherheit und Gesundheit von Frauen in Deutschland. Eine Repräsentative Untersuchung zu Gewalt Gegen Frauen in Deutschland.

[11] Schubert C.C., Punamäki R.-L. (2011). Mental health among torture survivors: Cultural background, refugee status and gender. Nord. J. Psychiatry.

[12] Alemi Q., James S., Siddiq H., Montgomery S. (2015). Correlates and predictors of psychological distress among Afghan refugees in San Diego County. Int. J. Cult. Ment. Health.

[13] Hollander A.-C., Bruce D., Burström B., Ekblad S. (2011). Gender-related mental health differences between refugees and non-refugee immigrants—A cross-sectional register-based study. BMC Public Health.

[14] Rizkalla N., Arafa R., Mallat N.K., Soudi L., Adi S., Segal S.P. (2020). Women in refuge: Syrian women voicing health sequelae due to war traumatic experiences and displacement challenges. J. Psychosom. Res..

[15] Ozel S., Yaman S., Kansu-Celik H., Hancerliogullari N., Balci N., Engin-Ustun Y. (2018). Obstetric Outcomes among Syrian Refugees: A Comparative Study at a Tertiary Care Maternity Hospital in Turkey. Rev. Bras. Ginecol. Obs. RBGO Gynecol. Obstet..

[16] Malebranche M., Nerenberg K., Metcalfe A., Fabreau G.E. (2017). Addressing vulnerability of pregnant refugees. Bull. World Health Organ..

[17] Bozorgmehr K., Biddle L., Preussler S., Mueller A., Szecsenyi J. (2018). Differences in pregnancy outcomes and obstetric care between asylum seeking and resident women: A cross-sectional study in a German federal state, 2010–2016. BMC Pregn. Childbirth.

[18] Deacon Z., Sullivan C. (2009). Responding to the Complex and Gendered Needs of Refugee Women. J. Women Soc. Work.

[19] Hammer L., Arango D., Damboeck J., Rubiano E., Villacres D. (2019). Addressing the Needs of Women and Girls in Contexts of Forced Displacement: Experiences from Operations.

[20] Özvarış Ş.B., Hricak H. (2019). Safe spaces for women in challenging environments. Lancet Glob. Health.

[21] Khamphakdy-Brown S., Jones L.N., Nilsson J.E., Russell E.B., Klevens C.L. (2006). The Empowerment Program: An Application of an Outreach Program for Refugee and Immigrant Women. J. Ment. Health Couns..

[22] Sabri B., Njie-Carr V.P.S., Messing J.T., Glass N., Brockie T., Hanson G., Case J., Campbell J.C. (2019). The weWomen and ourCircle randomized controlled trial protocol: A web-based intervention for immigrant, refugee and indigenous women with intimate partner violence experiences. Contemp. Clin. Trials.

[23] Gren L., Frost C.J., Benson S., Jaggi R. (2015). Improving refugee women’s health: Building self-efficacy through monthly health workshops. Ann. Glob. Health.

[24] Van Der Kloof A., Bastiaanssen J., Martens K. (2014). Bicycle lessons, activity participation and empowerment. Case Stud. Transp. Policy.

[25] Ohanesian A. (2019). Picturing health: Health services in refugee camps are helping South Sudanese women tell their stories of sexual violence. Lancet.

[26] Listo R. (2018). Preventing violence against women and girls in refugee and displaced person camps: Is energy access the solution?. Energy Res. Soc. Sci..

[27] Grabska K. (2011). Constructing ‘modern gendered civilised’ women and men: Gender-mainstreaming in refugee camps. Gend. Dev..

[28] Karin S., Chowdhury M.A., Hasnat M.A., Tarin N.J. (2020). Status of Rohingya in Refugee Camps of Bangladesh: A Review Study. OALib.

[29] Stark L., Robinson M.V., Seff I., Gillespie A., Colarelli J., Landis D. (2021). The Effectiveness of Women and Girls Safe Spaces: A Systematic Review of Evidence to Address Violence Against Women and Girls in Humanitarian Contexts. Trauma Violence Abus..

[30] Krause U. (2014). Analysis of Empowerment of Refugee Women in Camps and Settlements. J. Intern. Displac..

[31] Rabe H. (2015). Effektver Schutz vor Geschlechtsspezifischer Gewalt—Auch in den Flüchtlingsunterkünften.

[32] Nikendei C., Huhn D., Adler G., von Rose P.B., Eckstein T.M., Fuchs B., Gewalt S.C., Greiner B., Günther T., Herzog W. (2017). Entwicklung und Implementierung einer Medizinischen Ambulanz in einer Erstaufnahmeeinrichtung fur Asylsuchende des Landes Baden-Wurttemberg [Development and implementation of an outpatient clinic at an initial reception centre for asylum seekers in the German federal state of Baden-Wuerttemberg]. ZEFQ.

[33] Manok N., Huhn D., Kohl R.M., Ludwig M., Schweitzer J., Kaufmann C., Terhoeven V., Ditzen B., Herpertz S.C., Herzog W. (2017). Ambulanz für Geflüchtete mit Traumafolgestörungen und psychischen Belastungen in einer Landeserstaufnahmeeinrichtung: Entwicklung, Implementierung und Patientenspektrum [Outpatient clinic for refugees with posttraumatic disorders and mental burdens in a state reception center]. Psychotherapeut.

[34] Nikendei C., Kindermann D., Brandenburg-Ceynowa H., Derreza-Greeven C., Zeyher V., Junne F., Friederich H.C., Bozorgmehr K. (2019). Asylum seekers’ mental health and treatment utilization in a three months follow-up study after transfer from a state registration-and reception-center in Germany. Health Policy.

[35] Wahedi K., Nöst S., Bozorgmehr K. (2017). Die Gesundheitsuntersuchung von Asylsuchenden: Eine bundesweite Analyse der Regelungen in Deutschland. § 62 Asylverfahrensgesetz [Health examination of asylum seekers: A nationwide analysis of state policies in Germany. § 62 of the asylum law. Bundesgesundheitsbl.

[36] Helfferich C. (2011). Die Qualität qualitativer Daten. Manual für die Durchführung Qualitativer Interviews.

[37] IBM Corp (2016). IBM SPSS Statistics for Windows, Version 24.0.

[38] VERBI Software (2012). MAXQDA. Software for Qualitative Data Analysis.

[39] Mayring P. (2010). Qualitative Inhaltsanalyse. Grundlagen und Techniken.

[40] Strauss A., Corbin J. (1998). Basics of Qualitative Research Techniques and Procedures for Developing Grounded Theory.

[41] Agaibi C.E., Wilson J.P. (2005). Trauma, PTSD, and Resilience: A Review of the Literature. Trauma Violence Abus..

[42] Farhood L.F., Richa H., Massalkhi H. (2014). Group Mental Health Interventions in Civilian Populations in War-Conflict Areas: A Lebanese Pilot Study. J. Transcult. Nurs..

[43] Alemi Q., James S., Montgomery S. (2016). Contextualizing Afghan refugee views of depression through narratives of trauma, resettlement stress, and coping. Transcult. Psychiatr..

[44] Markova V., Sandal G.M. (2016). Lay Explanatory Models of Depression and Preferred Coping Strategies among Somali Refugees in Norway. A Mixed-Method Study. Front. Psychol..

[45] Braun-Lewensohn O., Abu-Kaf S., Al-Said K. (2019). Women in Refugee Camps: Which Coping Resources Help Them to Adapt?. Int. J. Environ. Res. Public Health.

[46] Asgary R., Segar N. (2011). Barriers to Health Care Access among Refugee Asylum Seekers. J. Health Care Poor Underserved.

[47] Zehetmair C., Tegeler I., Kaufmann C., Klippel A., Reddemann L., Junne F., Herpertz S.C., Friederich H.C., Nikendei C. (2019). Stabilizing Techniques and Guided Imagery for Traumatized Male Refugees in a German State Registration and Reception Center: A Qualitative Study on a Psychotherapeutic Group Intervention. J. Clin. Med..

[48] Zehetmair C., Nagy E., Leetz C., Cranz A., Kindermann D., Reddemann L., Nikendei C. (2020). Self-Practice of Stabilizing and Guided Imagery Techniques for Traumatized Refugees via Digital Audio Files: Qualitative Study. J. Med Internet Res..

[49] Zehetmair C., Zeyher V., Cranz A., Ditzen B., Herpertz S.C., Kohl R.M., Nikendei C. (2021). A Walk-In Clinic for Newly Arrived Mentally Burdened Refugees: The Patient Perspective. Int. J. Environ. Res. Public Health.

[50] Priebe K., Giacco D., El-Nagib R. (2016). Public Health Aspects of Mental Health among Migrants and Refugees: A Review of the Evidence on Mental Health Care for Refugees, Asylum Seekers and Irregular Migrants in the WHO European Region (Health Evidence Network (HEN) Synthesis Report 47).

[51] Bartolomei L., Eckert R., Pittaway E. (2014). “What happens there… follows us here”: Resettled but Still at Risk: Refugee Women and Girls in Australia. Refuge.

[52] Olivius E. (2013). (Un)Governable Subjects: The Limits of Refugee Participation in the Promotion of Gender Equality in Humanitarian Aid. J. Refug. Stud..

[53] Kreitzer L. (2002). Liberian refugee women: A qualitative study of their participation in planning camp programmes. Int. Soc. Work..

[54] Hebebrand J., Anagnostopoulos D., Eliez S., Linse H., Pejovic-Milovancevic M., Klasen H. (2016). A first assessment of the needs of young refugees arriving in Europe: What mental health professionals need to know. Eur. Child Adolesc. Psychiatry.

[55] Bronstein I., Montgomery P. (2011). Psychological Distress in Refugee Children: A Systematic Review. Clin. Child Fam. Psychol. Rev..

[56] Brooks L.A., Manias E., Bloomer M.J. (2019). Culturally sensitive communication in healthcare: A concept analysis. Collegian.

[57] Bernal G., Jiménez-Chafey M.I., Rodríguez M.M.D. (2009). Cultural adaptation of treatments: A resource for considering culture in evidence-based practice. Prof. Psychol. Res. Pract..

[58] Gavranidou M., Abdallah-Steinkopff B. (2007). Brauchen Migrantinnen und Migranten eine andere Psychotherapie?. Psychotherapeutenjournal.

[59] Hutnik N., Gregory J. (2008). Cultural sensitivity training: Description and evaluation of a workshop. Nurse Educ. Today.

[60] Nesterko Y., Glaesmer H. (2016). Migration und Flucht als Prozess. Trauma Gewalt.

[61] American Psychiatric Association (2013). Diagnostic and Statistical Manual of Mental Disorders (DSM-5).

[62] Chatzea V.-E., Sifaki-Pistolla D., Vlachaki S.-A., Melidoniotis E., Pistolla G. (2018). PTSD, burnout and well-being among rescue workers: Seeking to understand the impact of the European refugee crisis on rescuers. Psychiatr. Res..

[63] Woo M.-J., Kim D.-H. (2020). Factors Associated With Secondary Traumatic Stress Among Nurses in Regional Trauma Centers in South Korea: A Descriptive Correlational Study. J. Emerg. Nurs..

[64] Lusk M., Terrazas S. (2015). Secondary Trauma among Caregivers Who Work With Mexican and Central American Refugees. Hisp. J. Behav. Sci..

[65] Kindermann D., Jenne M.P., Schmid C., Bozorgmehr K., Wahedi K., Junne F., Szecsenyi J., Herzog W., Nikendei C. (2019). Motives, experiences and psychological strain in medical students engaged in refugee care in a reception center—A mixed-methods approach. BMC Med Educ..

[66] Plakas C. (2018). Burnout, Compassion Fatigue, and Secondary Traumatic Stress among Humanitarian Aid Workers in Jordan. https://www.researchgate.net/publication/328685237.

[67] Akinsulure-Smith A.M., Espinosa A., Chu T., Hallock R. (2018). Secondary Traumatic Stress and Burnout among Refugee Resettlement Workers: The Role of Coping and Emotional Intelligence. J. Trauma. Stress.

[68] Isawi D.T., Post P.B. (2020). Self-Efficacy of Counselors Working With Refugees. Adultspan J..

[69] Kim Y.J. (2017). Secondary Traumatic Stress and Burnout of North Korean Refugees Service Providers. Psychiatry Investig..

[70] Wirth T., Mette J., Prill J., Harth V., Nienhaus A. (2019). Working conditions, mental health and coping of staff in social work with refugees and homeless individuals: A scoping review. Health Soc. Care Community.

[71] Hagaman A.K., Wutich A. (2017). How Many Interviews Are Enough to Identify Metathemes in Multisited and Cross-cultural Research? Another Perspective on Guest, Bunce, and Johnson’s (2006) Landmark Study. Field Methods.

